# Training in neuropsychiatry: views of early career psychiatrists from across the world

**DOI:** 10.1192/bjb.2023.32

**Published:** 2024-04

**Authors:** Rosa Molina-Ruiz, Yukako Nakagami, Sabrina Mörkl, Martin Vargas, Mohammadreza Shalbafan, Jane Pei-Chen Chang, Yugesh Rai, Champion T. Seun-Fadipe, Gamze Erzin, Firoz Kazhungil, Pablo Vidal, Sawitri Brihastami, Eren Yıldızhan, Tanay Maiti, Ilya Fedotov, Irena Rojnic-Palavra, Toru Horinouchi, Vasanth Renganathan, Mariana Pinto da Costa

**Affiliations:** 1 Hospital Clínico Universitario San Carlos, Madrid, Spain; 2 Kyoto University Graduate School of Medicine, Kyoto, Japan; 3 Medical University of Graz, Graz, Austria; 4 Universidad de Valladolid, Valladolid, Spain; 5 Iran University of Medical Sciences, Tehran, Iran; 6 Institute for Cognitive Sciences Studies, Tehran, Iran; 7 China Medical University Hospital, Taichung, Taiwan; 8 Essex Partnership University NHS Foundation Trust, Colchester, UK; 9 Nottinghamshire Healthcare NHS Trust, Nottingham, UK; 10 Diskapi Training and Research Hospital, Ankara, Turkey; 11 NMC Royal Hospital, Abu Dhabi, United Arab Emirates; 12 Hospitalario Universitario A Coruña, A Coruña, Spain; 13 Universitas Airlangga, Surabaya, Indonesia; 14 Bakirkoy Mazhar Osman Research and Training Hospital for Psychiatry, Istanbul, Turkey; 15 South Yorkshire NHS Foundation Trust, Dewsbury, UK; 16 Ryazan State Medical University, Ryazan, Russia; 17 University Psychiatric Hospital Sveti Ivan, Zagreb, Croatia; 18 Hokkaido University Graduate School of Medicine, Sapporo, Japan; 19 Fortis Malar Hospital, Chennai, India; 20 Institute of Psychiatry, Psychology & Neuroscience, King's College London, London, UK; 21 Institute of Biomedical Sciences Abel Salazar, University of Porto, Porto, Portugal

**Keywords:** Neuropsychiatry, neuroscience, clinical neuroscience, education, training

## Abstract

**Background:**

Training and practice in neuropsychiatry varies across the world. However, little is known about the experiences and opinions of early career psychiatrists (ECPs) across different countries regarding neuropsychiatry.

**Aims and method:**

To investigate neuropsychiatry training experiences, practices and opinions among ECPs across different countries. An online survey was distributed to ECPs in 35 countries across the world.

**Results:**

A total of 522 participants took part in this study. Responses show that neuropsychiatry is integrated to a variable extent in psychiatric training curricula across the world. Most respondents were not aware of the existence of neuropsychiatric training or of neuropsychiatric units. Most agreed that training in neuropsychiatry should be done during or after the psychiatry training period. Lack of interest among specialty societies, lack of time during training, and political and economic reasons are regarded as the main barriers.

**Clinical implications:**

These findings call for an improvement in the extent and in the quality of neuropsychiatry training across the world.

The advances of neurosciences in mental healthcare underline the importance of integrating neuropsychiatry and clinical neuroscience in the training of mental health professionals.^1^ There is increasing international recognition that the future professional role of a psychiatrist will demand a greater knowledge of brain functions than that which is currently taught.^2^ The ability of tomorrow's psychiatrists to give their patients the benefits of a neuroscience-enhanced understanding of psychopathology, diagnosis and treatment is related to the capacity of postgraduate training programmes to provide adequate training in basic, applied and clinical neuroscience.^3^ This training should be understood inclusively, in conjunction with the humanistic aspects of psychiatry and not in a dualistic manner.

Importantly, psychiatric diagnosis relies on cross-sectional biobehavioural dimensions that are common to multiple categories of heterogeneous disorders. Research domain criteria (RDoC)^4^ have been proposed to advance the research into aetiology and pathophysiology as well as the development of new treatments. This advancement relies on adequate knowledge not only of the psychopathology of mental disorders but also of their neuropsychiatric and neuroscientific foundations.

The World Health Organization (WHO) considers neuropsychiatric disorders to be the most important causes of disability worldwide,^5^ and the psychiatric complications of neurological diseases are considered to cause major distress and burden to patients and their carers.^6^ However, the majority of hospital-based neuropsychiatric disease is still being treated by liaison psychiatrists, old age psychiatrists, general adult psychiatrists and, often reluctantly, by medical teams. This is due not only to a lack of specialist neuropsychiatrists but also to the inadequate training for hospital specialists,^6^ which is an international problem.

Training, practice and continuing professional development in neuropsychiatry vary across different countries. There is a substantial global recognition of the need to incorporate this field into the postgraduate training of psychiatrists.^3,6–8^ However, the definition of neuropsychiatry is still unclear in different countries, varying from a more global concept of neuropsychiatry^9,10^ and clinical neuroscience to the basic neurological concepts that are taught during medical school or postgraduate training. Several related terms, such as ‘biological psychiatry’, ‘organic psychiatry’, ‘cognitive neurosciences’ and ‘behavioural neurology’, have perpetuated this confusion.^6^ A better definition and a specific curriculum are needed. This differentiation of neuropsychiatry as a specialty is also supported by numerous textbooks in this area.^11^

As regards neuropsychiatry as part of the psychiatrist's training curricula,^12^ it seems that most training has been placed in the neurology training period, without really developing a specific curriculum for neuropsychiatry. Although psychiatry and neurology both deal with brain diseases, there is a need to have a combined neuropsychiatric approach, which requires the delineation of a neuropsychiatric territory and the competencies that should be achieved.^13^

This is not a new concept, as previous surveys have been conducted on both neuroscience and neuropsychiatry curricula. For example, in Australia and the USA this issue has been addressed and results have pointed to the necessity and readiness of psychiatry training programmes to provide increased neuroscience and neuropsychiatry education.^6,14^ Importantly, an international curriculum for neuropsychiatry and behavioural neurology has been developed describing in detail the objectives of training in neuropsychiatry and the key competencies that should be achieved.^3,13^ Other studies have investigated the content of the training provided in psychiatry programmes, reporting that in 80% of the countries studied, a placement in a non-psychiatric specialty such as neurology or internal medicine was mandatory, although it was not always considered enough or well-structured.^15,16^ A survey of 100 trainees in England investigated the attitudes of neurologists and psychiatrists to strengthening their links, showing that psychiatrists, in general, were even more aware of links between the two specialties.^17^ A survey in Australia involving 47 trainees who had worked in the neuropsychiatry unit at the Royal Melbourne Hospital reported very positive feedback, particularly on the breadth of clinical presentations, research and educational opportunities.^14^ Other studies that assessed the attitudes of trainees, chief residents or residency programme directors in the USA described broad support for neuroscience education and a desire to increase its representation in psychiatric training.^18–20^

Some countries, such as the USA,^11,21^ and Australia^22^ have developed specific curricula for neuropsychiatry trainees, recognising neuropsychiatry as a subspecialty. In the UK, while neuropsychiatry is not formally endorsed as a subspecialty by the Gneral Medical Council (GMC), advanced training and education in neuropsychiatry are available through specialist training posts and through the MSc in Clinical Psychiatry provided by the University of Birmingham and King's College London.^23^

However, to date little is known about the opinions of early career psychiatrists (ECPs) around the world on their neuropsychiatry training experiences. Their opinions regarding barriers to neuropsychiatry training and practice have not been addressed. This study aimed to investigate the views of ECPs on their training experiences and the barriers to neuropsychiatry training and practice across different countries around the world.

## Method

### Study design

This online survey was distributed to ECPs in 35 countries worldwide (Australia, Austria, United Arab Emirates, Brazil, Chile, Croatia, Ecuador, Egypt, El Salvador, France, Germany, Guinea-Bissau, Indonesia, Iran, Italy, Japan, Kosovo, North Macedonia, Malaysia, Nepal, Nigeria, Philippines, Portugal, Russia, Sri Lanka, Slovenia, Spain, Switzerland, Sweden, South Africa, Taiwan, Thailand, Turkey, the UK and USA). In this study ECPs is used to refer to psychiatric trainees and psychiatrists in their first 7 years of working as consultants.

### Study instrument

The questionnaire was developed in collaboration with the Spanish Clinical Neuroscience Section, the European Federation of Psychiatric Trainees (EFPT) and the Section of Early Career Psychiatrists of the World Psychiatric Association (WPA).

The questionnaire was developed in English and asked participants about their training opportunities in neuropsychiatry. The questions were grouped into four main domains: (I) sociodemographic details; (II) neuropsychiatry training experience; (III) barriers to neuropsychiatry training; and (IV) opinions regarding neuropsychiatry training/practice.

The following description of the term ‘neuropsychiatry' was included at the beginning of the survey: ‘Neuropsychiatry in a broad sense refers to the clinical study, evaluation and treatment of brain–behavior relationships as revealed through the psychiatric manifestations of neurological disorders and the neurobiology of psychiatric disorders’.^3^

In the literature the terms ‘neuropsychiatry', ‘neuroscience' and ‘clinical neuroscience' are used to refer to components of neuropsychiatry training, and in this article we retain the term.

### Participants

Participants for this study met the following inclusion criteria: qualified medical doctor training in psychiatry, or psychiatrist in the first 7 years of working as a consultant.

### Data collection

The questionnaire was distributed in each country through the study's national representatives, who were responsible for contacting their national psychiatry associations or scientific societies.

Participants were contacted by email and an online questionnaire was sent via Survey Monkey with a URL to a survey webpage. Information about the study was provided on the webpage. Responses were collected between 1 January to 31 March 2018.

### Data analysis

Data were analysed using SPSS version 17.0 for Windows. Data are presented as means with standard deviations (s.d.) or frequencies and percentages, as appropriate. Missing data were omitted on an analysis-by-analysis basis and valid percentages are reported.

### Consent and study approvals

By responding to the questionnaire, participants provided informed consent for their anonymous responses to be used in this study.

The authors assert that all procedures contributing to this work comply with the ethical standards of the relevant national and institutional committees on human experimentation and with the Helsinki Declaration of 1975, as revised in 2008.

This study did not involve a prospective evaluation or involve animals or vulnerable participants (e.g. patients). The research did not pose risks, harm or disadvantage to the participants, as it assessed anonymous data from competent adults only. According to the procedures in comparable cases, ethical approval was therefore not required.

## Results

### Sociodemographic data

A total of 522 participants took part in this study and 509 fully completed the questionnaire. Sociodemographic data are shown in Table 1. The mean age was 33.5 years (s.d. = 6.1 years); 53% (*n* = 275) were female and 46% (*n* = 240) male. Most responses were collected from Europe (*n* = 234, 45%) and Asia (*n* = 217, 42%). Only 3.5% of the sample were currently specialised in neuropsychiatry.

**Table 1:** Sociodemographic data for respondents

Variable	Frequency, *n*	Proportion, %
Gender		
Female	275	52.9
Male	240	46.2
Continent		
Asia	217	41.7
Europe	234	45.0
Africa	43	8.3
America	12	2.3
Australia	3	0.6
Current specialty		
Psychotherapy	25	5.0
General adult psychiatry	171	32.9
Neuropsychiatry	18	3.5
Forensic	5	1.0
Just training	290	56.0

The majority of the respondents (*n* = 432, 83%) reported that they had a mandatory rotation in neurology; fewer (*n* = 71, 14%) said that this rotation was not mandatory. In countries where this rotation was mandatory, its duration ranged from 1 to 6 months (mean 2.6, s.d. = 1.4).

### Neuropsychiatry units and training experiences

A quarter of the respondents (*n* = 139, 27%) reported that they knew of a neuropsychiatry unit in their city, but the majority (*n* = 375, 72%) of the participants did not answer this question or answered ‘no’ or ‘I don't know’. Nearly half of the participants (*n* = 241, 46%) reported that a clinical neuropsychiatry rotation was not mandatory, and only a few (*n* = 82, 16%) reported neuropsychiatry as mandatory (Table 2).

**Table 2:** Respondents’ description of their neuropsychiatry training

Variable	Frequency or mean	% or s.d.
Neurology as a mandatory training		
Yes	432	83.0
No	71	13.7
Duration of neurology rotation, months	2.66	1.43
Aware of any neuropsychiatry unit		
Yes	139	26.7
No	288	55.0
Don't know	87	16.7
Neuropsychiatry as a mandatory rotation		
No	241	46.3
Yes	82	15.8
Aware of neuropsychiatry training		
No	333	64.0
Master	37	7.1
Ongoing seminars, journal clubs, etc.	35	6.7
Fellowships	30	5.8
Interested in further neuropsychiatry training abroad		
Yes	117	22.5
No	312	60.0
Don't know	2	0.4
Expected to involve in research in neuropsychiatry		
Yes	304	58.5
No	201	38.7
Expected PhD in neuropsychiatry		
Yes	138	26.5
No	366	70.4
Most requested field to go abroad for		
Clinical management strategies	141	27.1
Neuroscience	75	14.4
Psychotherapy, clinical management strategies	66	12.7
Is neuropsychiatry or clinical neuroscience promoted?		
Yes	27	5.2
No	109	21.0

The majority (*n* = 333, 64%) of participants were not aware of any neuropsychiatry postgraduate training in clinical neuropsychiatry in their countries; many (*n* = 312, 60%) were not interested in going abroad for further training in neuropsychiatry. Some (*n* = 304, 58%) reported that they were expected to get involved in research projects in neuropsychiatry and fewer (*n* = 138, 26%) were expected to conduct a PhD in neuropsychiatry or clinical neuroscience. Among the main fields to go abroad for during the postgraduate training period, some (*n* = 75, 14%) rated neuroscience among the most requested fields (neuropsychiatry was not included as a choice in this question) (Table 2). Others requested fields such as psychotherapy (*n* = 66, 13%) and clinical management strategies (*n* = 141, 27%).

### Barriers to and implementation of neuropsychiatry training

Lack of interest among specialty societies, lack of time during postgraduate training, and political and economic limitations were the main barriers to the implementation of neuropsychiatry in training (Table 3).

**Table 3:** Respondents’ opinions regarding neuropsychiatry training

Variables	Frequency	%
Obstacles to implementation of neuropsychiatry		
Lack of time during residency	211	40.6
Economic limitations	229	44.0
Lack of interest among the specialty societies	239	46.0
Political reasons	72	13.9
Don't know	5	1.0
Best way of developing educational resources		
Interdisciplinary meetings	211	40.6
Training during residency	304	58.5
Master during residency	68	13.1
Master post residency	107	20.6
Common neuroscience training period during first years of specialisation	173	33.3
Best way of implementation of neuropsychiatry training		
During residency (without increasing total duration)	333	64.0
During residency (with increasing total duration)	93	17.9
After residency	130	25.0
Before residency	41	7.9
Master after residency	70	13.5
Only ongoing seminars	30	5.8

The preferred timings on when best to provide neuropsychiatry training were during and after the training period (*n* = 333, 64% and *n* = 130, 24% respectively). However, most respondents who chose ‘during training’ preferred this to be done by increasing the duration of training (*n* = 93, 18%), compared with ‘without increasing duration’ (*n* = 33, 6%). Just a few (*n* = 41, 8%) thought it should be implemented before psychiatry training.

On a Likert scale from 1 to 10, most agreed on the priority for a better relationship between neurology and psychiatry (mean 8.4, s.d. = 1.8), followed by the necessity for further training in neuropsychiatry (mean 7.7, s.d. = 1.9), as well as the need for further training in research into neuropsychiatry (mean 7.7, s.d. = 1.9) and research into neuroscience (mean 7.4, s.d. = 2.1) (Tables 3 and 4).

**Table 4:** Opinions regarding neuropsychiatry training/practice (Likert scale of 1–10, where 10 is maximum agreement)

Questions	*N*	Mean	s.d.
How would you rate your training in neuropsychiatry?	491	4.32	2.18
How would you rate the necessity for further training in neuropsychiatry?	512	7.78	1.95
How would you rate your training in research in neuroscience?	499	3.86	2.42
How would you rate the necessity for further training in research in neuroscience?	505	7.48	2.16
How good is the relationship between neurologists and psychiatrists in your country?	511	5.14	2.22
How would you rate the necessity for a better relationship between neurology and psychiatry?	512	8.43	1.84

## Discussion

### Key findings

This study provides further evidence of an ongoing unmet need in the provision of pathways to gaining neuropsychiatry practice and raises awareness of the willingness for further training in neuropsychiatry among ECPs in different parts of the world. Most respondents were not aware of any neuropsychiatric training in their curricula or of local neuropsychiatric units. Most agreed that training in neuropsychiatry training should be done during the psychiatry training period or after training. Of those who considered that training in neuropsychiatry should take place during psychiatry training, most felt this should be achieved by increasing the duration of training.

Neuropsychiatry education is not integrated into psychiatric training in most countries. The majority of the participants reported that learning about neuropsychiatry is not included in their psychiatric curricula as a mandatory rotation. Lack of interest among specialty societies, lack of time during postgraduate training, political and economic reasons are regarded as main barriers.

### Comparison with the literature

Regarding the most general sociodemographic details of this survey, we know that psychiatric training has changed across different countries in recent decades and that several subspecialties, such as psychotherapy and addiction, have been integrated into psychiatric curricula as mandatory rotations, not only in high-income but also in low- and middle-income countries on different continents;^24-27^ this has not been the case for neuropsychiatry. This finding is consistent with previous reports from the UK, in which 73% of the sample had no previous clinical training experience in neuropsychiatry, but 74% expressed a wish specifically to train in this subject and 87% stated a desire for a clearly defined curriculum.^28,29^ In the UK the RCPsych curriculum was amended to enhance neuroscience and neuropsychiatry (MRCPsych Syllabus amendment^23^). According to a survey of psychiatry residency programme directors in the USA, 64% and 60% agreed that knowledge of neuropsychiatry and knowledge of psychiatric neuroscience respectively were either very important or critically important for providing excellent care.^20^

Regarding the most general sociodemographic details of this survey, we know that psychiatric training has changed across different countries in recent decades and that several subspecialties, such as psychotherapy and addiction, have been integrated into psychiatric curricula as mandatory rotations, not only in high-income but also in low- and middle-income countries on different continents;^24-27^ this has not been the case for neuropsychiatry. This finding is consistent with previous reports from the UK, in which 73% of the sample had no previous clinical training experience in neuropsychiatry, but 74% expressed a wish specifically to train in this subject and 87% stated a desire for a clearly defined curriculum.^28,29^ In the UK the RCPsych curriculum was amended to enhance neuroscience and neuropsychiatry (MRCPsych Syllabus amendment^23^). According to a survey of psychiatry residency programme directors in the USA, 64% and 60% agreed that knowledge of neuropsychiatry and knowledge of psychiatric neuroscience respectively were either very important or critically important for providing excellent care.^20^

In the subsample from Nepal in our survey, two-thirds of respondents stated that there is no neuropsychiatry training during psychiatry training in their country. However, in a study on neuropsychiatry training in Nepal the majority of ECPs (85%) stated that neurology is integrated into their training course as a mandatory rotation.^29^ These results highlight the need for specific training in neuropsychiatry beyond the neurology rotation.

As regards barriers to neuropsychiatry training and views on its implementation, more than half of the participants (58.5%) expressed interest in participating in neuropsychiatric research projects. The increasing interest of psychiatrists, particularly ECPs, has also been previously reported in Asia.^30^ This is consistent with other findings in Nepal concerning the need for future training in neuropsychiatry, where the median score on a ten-point Likert scale was 7.9.^29^ In two different USA nationwide surveys, most participants (chairs of psychiatry departments, psychiatrists and psychiatric trainees) agreed with the need for more neuroscience education.^18,20^ Similar results were reported in the UK.^28^

In addition, 24% of participants in our study stated that the best way to develop their knowledge in this field is the integration of neuropsychiatry training into the psychiatric training course: 18% by modifying the duration of postgraduate training and 6% without modifying it. Only 8% stated neuropsychiatry training should be done before postgraduate training. Of note, 25% considered it should be done after postgraduate training. This is a very interesting finding because, after the modification of psychiatric training courses as a specialty for medical graduate physicians, the duration of training in psychiatry has been increased from 4 to 5 years in almost all European countries (Table 1), including education on subspecialties such as addiction and psychotherapy.^25^ The knowledge, skills and competencies in neuropsychiatry could be included as core components of the main curriculum of training in psychiatry, as training modules as part of the main rotations, such as acute in-patient unit rotations or community psychiatry placements, which are the two main common periods of training of psychiatrists worldwide.^27^

Our findings suggest that lack of interest among specialty societies, lack of time during postgraduate training, and political and economic limitations are the main obstacles to the implementation of neuropsychiatry training. Similarly, previous studies in the USA^20^ found insufficient neuropsychiatry faculty (39%) and absence of neuroscience faculty (36%) to be the main barriers to the implementation of neuropsychiatry, even if three-quarters indicated that faculty resources were available in their departments. This means that even in the presence of several curriculum ideas that have been promulgated in this area,^3,13^ their adoption is not yet widespread and a lack of structural or faculty resources limits their adequate implementation.

With respect to the neuropsychiatry training experience, about three-quarters of our participants were not aware of neuropsychiatric clinics in their city or district. Although neuropsychiatric clinics have been developed and increased in recent decades,^31^ there is a lack of awareness about them among ECPs. This highlights the need for greater collaboration between neuropsychiatric clinics and psychiatric departments. Neuropsychiatry and brain and cognition clinics should pay attention to this and try to develop their collaboration with other psychiatrists.

### Strengths and limitations

To the best of our knowledge, this is the first international survey on the experiences, attitudes and challenges of ECPs regarding neuropsychiatry in their psychiatric training course and practice.

However, several limitations should be acknowledged. The concepts of neuropsychiatry and (clinical) neuroscience are still difficult to establish, since these are terms elusive in nature^9,32^ and understanding of them might vary across different countries. In some countries training in these fields might be limited to some training in neurology, basic neuroscience or just research into the biological components of mental disorders. We tried to unpick this in this survey by enquiring about specific rotations in neurology or neuropsychiatry. Participants’ responses might be affected by a lack of a similar understanding of what neuropsychiatry is, as well as differences in their prior exposure to neuropsychiatry.

Another limitation of our survey is the small sample from some world regions, such as the UK, USA, Australia and Africa, as the sample mainly came from Asian and European countries. This limits the generalisation of our findings.

### Implications of the findings for practice and research

There is a need for coherent and shared international consensus on what constitutes neuropsychiatry and what its training curriculum should entail. These findings can help guide scientific societies and professional organisations representing psychiatrists or responsible for setting the standards for postgraduate training in psychiatry towards further consensus on these concepts.

As shown by previous studies, a neuropsychiatry provision closely allied with neuroscience centres should be adopted widely to bring consistency across international neuropsychiatry training pathways to try to avoid undesirable variability and unmet needs.^10,33^

It has also been stated that training can improve clinical care^34^ but these need to be accompanied by systemic local organisational change to be effective.^35^

The fact that more than half of the participants showed an interest in neuropsychiatric research projects also emphasises a need for more structured and well-designed courses of neuropsychiatry in psychiatric training across the world. International associations such as the WPA could be involved in such processes. This might help psychiatric departments, particularly in low- and middle-income countries, to improve neuropsychiatric education.

Action is warranted to address our findings that the lack of interest among specialty societies, lack of time during postgraduate training, and political and economic limitations might be among the main obstacles to training in neuropsychiatry.

## Data Availability

The data is available from the corresponding author on reasonable request.

## About the authors

**Rosa Molina-Ruiz**, Department of Psychiatry, Hospital Clínico Universitario San Carlos, Madrid, Spain; **Yukako Nakagami**, Department of Psychiatry, Kyoto University Graduate School of Medicine, Kyoto, Japan. **Sabrina Mörkl**, Department of Psychiatry and Psychotherapeutic Medicine, Medical University of Graz, Graz, Austria. **Martin Vargas**, Facultad de Medicina, Hospital Universitario Río Hortega, Universidad de Valladolid, Valladolid, Spain. **Mohammadreza Shalbafan**, Mental Health Research Center, Psychosocial Health Research Institute (PHRI), Department of Psychiatry, School of Medicine, Iran University of Medical Sciences, Tehran, Iran; and Brain and Cognition Clinic, Institute for Cognitive Sciences Studies, Tehran, Iran. **Jane Pei-Chen Chang**, Department of Psychiatry, China Medical University Hospital, Taichung, Taiwan. **Yugesh Rai**, Essex Partnership University NHS Foundation Trust, Colchester, UK. **Champion T. Seun-Fadipe**, Nottinghamshire Healthcare NHS Trust, Nottingham, UK. **Gamze Erzin**, Department of Psychiatry, Diskapi Training and Research Hospital, Ankara, Turkey. **Firoz Kazhungil**, NMC Royal Hospital, Abu Dhabi, United Arab Emirates. **Pablo Vidal**, Hospitalario Universitario de A Coruña, A Coruña, Spain. **Sawitri Brihastami**, Department of Psychiatry, Faculty of Medicine, Universitas Airlangga, Surabaya, Indonesia. **Eren Yıldızhan**, Department of Neurology and Neurosurgery, Bakirkoy Mazhar Osman Research and Training Hospital for Psychiatry, Istanbul, Turkey. **Tanay Maiti**, South West Yorkshire NHS Foundation Trust, Dewsbury, UK. **Ilya Fedotov**, Ryazan State Medical University, Ryazan, Russia. **Irena Rojnic-Palavra**, University Psychiatric Hospital Sveti Ivan, Zagreb, Croatia. **Toru Horinouchi**, Department of Psychiatry and Neurology, Hokkaido University Graduate School of Medicine, Sapporo, Japan. **Vasanth Renganathan**, Fortis Malar Hospital, Chennai, India. **Mariana Pinto da Costa**, Institute of Psychiatry, Psychology & Neuroscience, King's College London, London, UK; and Institute of Biomedical Sciences Abel Salazar, University of Porto, Porto, Portugal.
